# Supplementary material for: The MAOA rs979605 Genetic Polymorphism Is Differentially Associated with Clinical Improvement Following Antidepressant Treatment between Male and Female Depressed Patients
Source: Int J Mol Sci. 2022 Dec 28;24(1):497. doi: 10.3390/ijms24010497 (PMC9820795; doi:10.3390/ijms24010497)
Supplement: Supplementary file 1 [file ijms-24-00497-s001.zip › supplementary_IJMS/STables_SFigure-captions_MAO_16-11-2022_IJMS.pdf]

Figure S1: LD associations between MAOA and MAOB variants

For the 6 genetic variants of *MAOA* (rs6323, rs2235186, rs979606, rs979605, and rs1137070) and *MAOB* (rs1799836), we show the LD plots constructed using HaploView (v4.2) in the whole population as well as in males and females separately. Values within boxes correspond to r^2 values. Variants with an r^2>80 are considered in strong LD. Block 1 defines the haplotype block for variants observed to be in strong LD.

Figure S2: 5HIAA/5HT transformations

For each of the non-transformed (top), square-root-transformed (middle), and $\log_2$-transformed (bottom) 5HIAA/5HT ratios at each time point (M0: left; M3: center; M6: right) are shown density histograms (left) and QQ plots (right). For histograms, 5HIAA/5HT ratios (x-axis) are plotted according to density (y-axis) in gray bars, while the normal distribution is shown as a black curve in histograms. *P*-values correspond to Shapiro-Wilk tests of normality.

Table S1: Associations of the HDRS score with *MAOA* rs979605 allelic subgroups

Results of mixed-effects models of the HDRS score in rs979605(A>G) allelic subgroups. Allelic subgroups were analyzed according to random XCI and escape from XCI status. HDRS was assessed using the Satterthwaite method. *: *P*<0.0125 to account for the 4 analyses. **den**: denominator; **df**: degrees of freedom; **MS**: mean squares; **num**: numerator; ***P***: *P*-value; **SS**: sum of squares; **XCI**: X-chromosome inactivation.

	Coding strategy	SS	MS	df (num)	df (den)	*F*	*P*	SS	MS	df (num)	df (den)	*F*	*P*
		A allele						*G allele*					
Age		17.26	17.26	1	175.27	0.64	0.43	4.56	4.56	1	295.99	0.14	0.70
Sex		208.15	208.15	1	187.82	7.66	**0.0062***	20.52	20.52	1	289.49	0.65	0.42
AD Class		124.54	31.14	4	181.84	1.15	0.34	123.44	30.86	4	303.02	0.98	0.42
Loss to follow-up (M1)	***Random XCI***	168.16	168.16	1	205.11	6.19	0.014	53.82	53.82	1	361.62	1.70	0.19
Smoking status	*Females (0, 1),*	37.72	18.86	2	177.10	0.69	0.50	21.14	10.57	2	296.73	0.33	0.72
Time	*Males (1)*	13198.22	4399.41	3	410.00	161.92	**<0.001***	29730.27	9910.09	3	694.95	313.17	**<0.001***
rs979605		51.88	51.88	1	192.39	1.91	0.17	25.48	25.48	1	287.37	0.81	0.37
Sex:Time		258.50	86.17	3	418.07	3.17	0.024	87.66	29.22	3	699.71	0.92	0.43
Time:rs979605		183.20	61.07	3	421.42	2.25	0.082	21.93	7.31	3	697.86	0.23	0.87
Age		17.26	17.26	1	175.27	0.64	0.43	4.56	4.56	1	295.99	0.14	0.70
Sex		105.15	105.15	1	165.49	3.87	0.051	0.11	0.11	1	287.29	0.0035	0.95
AD Class		124.54	31.14	4	181.84	1.15	0.34	123.44	30.86	4	303.02	0.98	0.42
Loss to follow-up (M1)	***Escape from XCI***	168.16	168.16	1	205.11	6.19	0.014	53.82	53.82	1	361.62	1.70	0.19
Smoking status	*Females (0, 1),*	37.72	18.86	2	177.10	0.69	0.50	21.14	10.57	2	296.73	0.33	0.72
Time	*Males (0)*	16946.01	5648.67	3	411.24	207.90	**<0.001***	29730.27	9910.09	3	694.95	313.17	**<0.001***
rs979605		23.52	23.52	1	177.45	0.87	0.35	25.48	25.48	1	287.37	0.81	0.37
Sex:Time		79.88	26.63	3	411.19	0.98	0.40	120.40	40.13	3	698.27	1.27	0.28
Time:rs979605		183.20	61.07	3	421.42	2.25	0.082	21.93	7.31	3	697.86	0.23	0.87

Table S2: Associations of the plasma 5HIAA/5HT ratio with *MAOB* rs1799836 without sex interactions

Results for mixed-effects models of the 5HIAA/5HT ratio without sex interactions included, assessed using the Satterthwaite method. *: P<0.05. **den**: denominator; **df**: degrees of freedom; **MS**: mean squares; **num**: numerator; ***P***: P-value; **SS**: sum of squares.

	SS	MS	df (num)	df (den)	F	P
Age	15.46	15.46	1	134.69	8.01	**0.0054***
Sex	0.71	0.71	1	135.70	0.37	0.55
AD class	42.27	10.57	4	135.74	5.47	**<0.001***
Smoking status	2.45	1.22	2	135.64	0.63	0.53
AD-naïve status	24.38	24.38	1	376.58	12.63	**<0.001***
Time	40.30	20.15	2	301.02	10.44	**<0.001***
rs1799836 dominance term	0.017	0.017	1	300.00	0.0086	0.93
rs1799836	11.59	11.59	1	135.00	6.00	**0.016***
Time:rs17998361	5.03	1.26	4	284.31	0.65	0.63
