# Supplementary figures and images for: The MAOA rs979605 Genetic Polymorphism Is Differentially Associated with Clinical Improvement Following Antidepressant Treatment between Male and Female Depressed Patients
Source: Int J Mol Sci. 2022 Dec 28;24(1):497. doi: 10.3390/ijms24010497 (PMC9820795; doi:10.3390/ijms24010497)

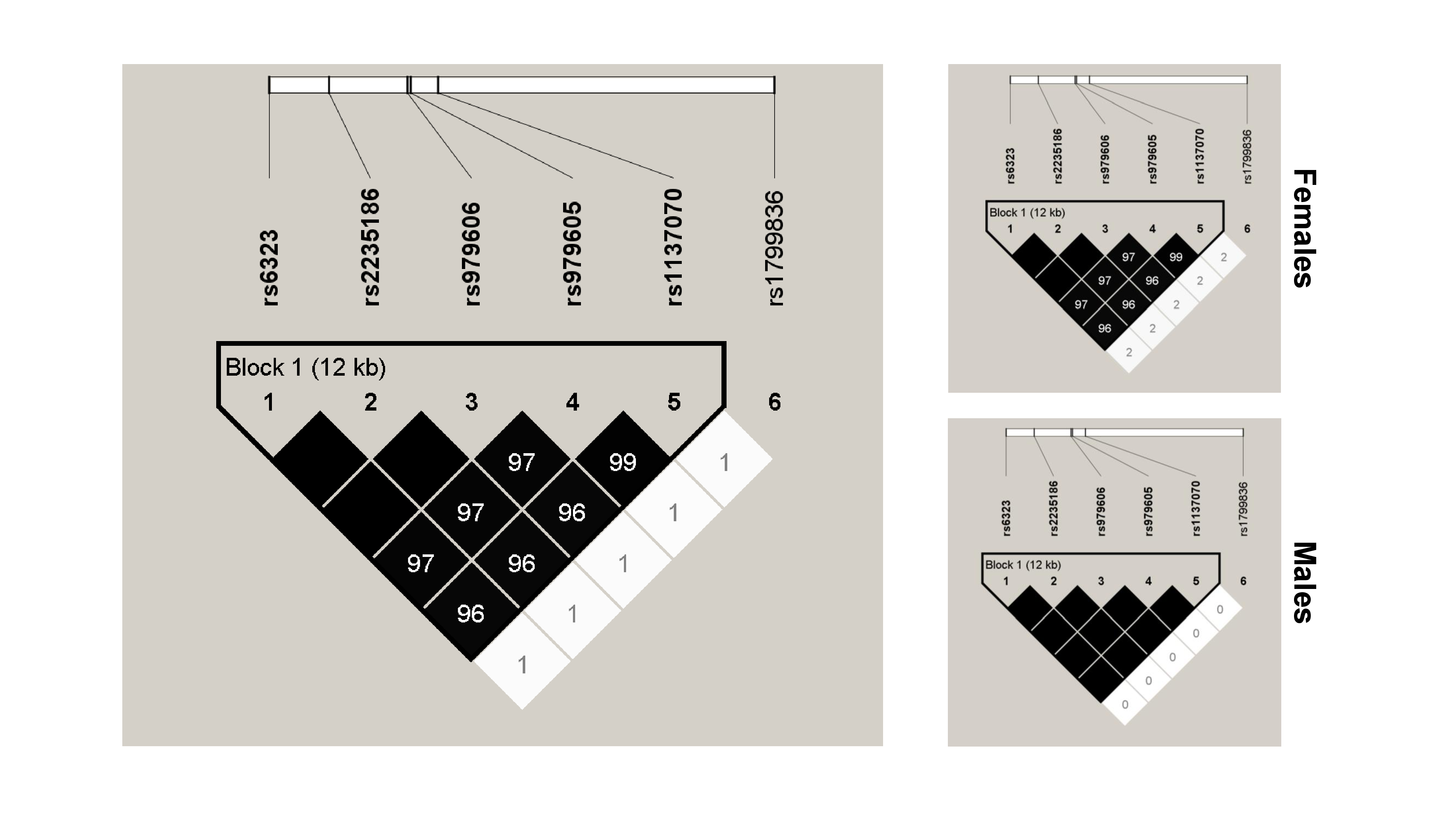

Supplement: Supplementary file 1 [file ijms-24-00497-s001.zip › supplementary_IJMS/FigureS1.tiff]

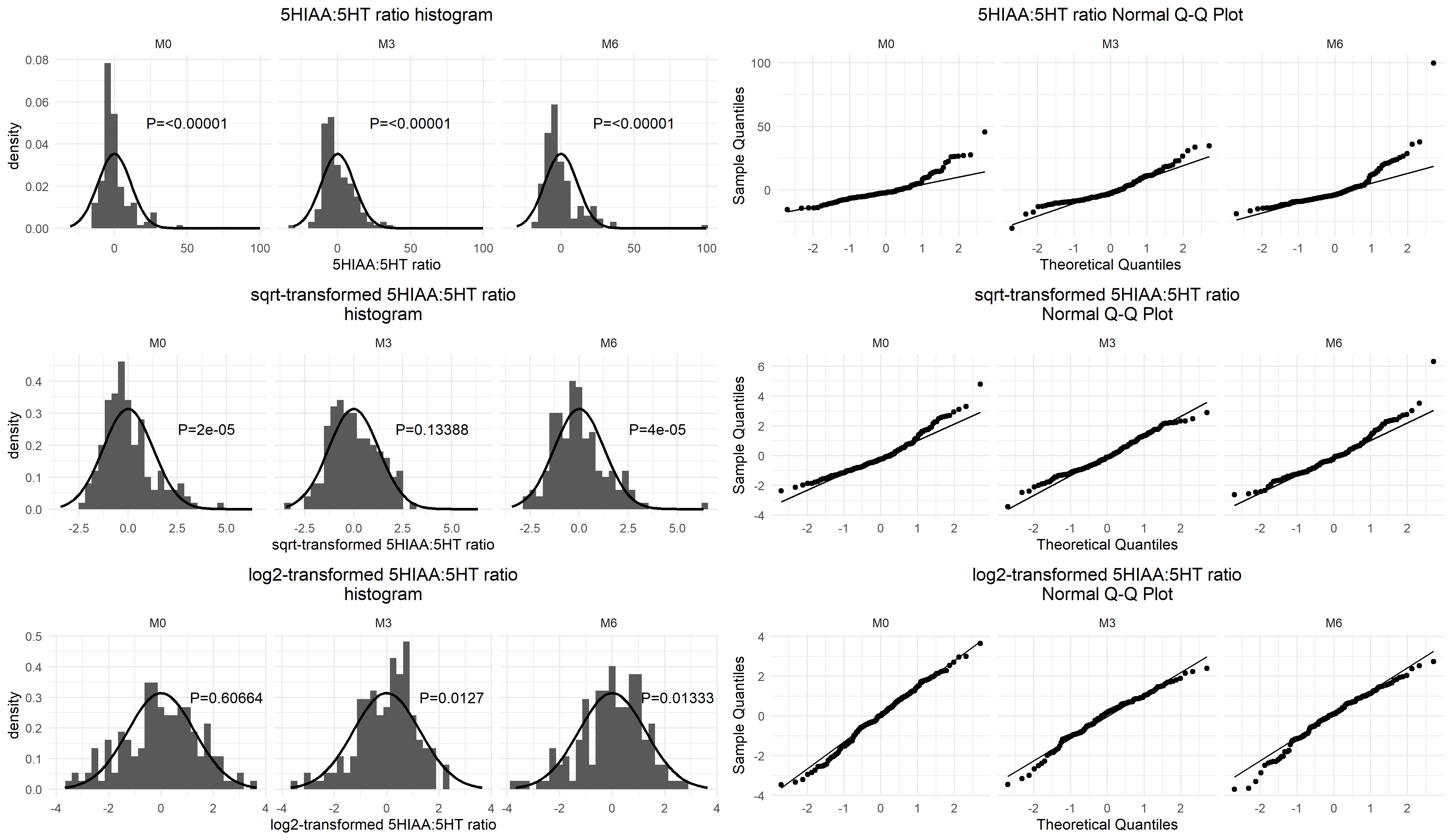

Supplement: Supplementary file 1 [file ijms-24-00497-s001.zip › supplementary_IJMS/FigureS2.tiff]
